# Development of High-Performance Ethanol Gas Sensors Based on La_2_O_3_ Nanoparticles-Embedded Porous SnO_2_ Nanofibers

**DOI:** 10.3390/s24216839

**Published:** 2024-10-24

**Authors:** Gen Li, Jian Hou, Muhammad Hilal, Hyojung Kim, Zhiyong Chen, Yunhao Cui, Jun-Hyun Kim, Zhicheng Cai

**Affiliations:** 1 College of Computer Science, Chengdu University, Chengdu 610106, China; ligen@cdu.edu.cn (G.L.); 2 School of Intelligent Manufacturing, Luoyang Institute of Science and Technology, Luoyang 471023, China; jhou@lit.edu.cn (J.H.); 1854@163.com (Z.C.); 3 Department of Semiconductor System Engineering, Sejong University, 209 Neungdong-ro, Gwangjin-gu, Seoul 05006, Republic of Korea; hilal1991@sejong.ac.kr (M.H.); hyojungkim0912@sejong.ac.kr (H.K.); 4 School of Mechatronics Engineering, Henan University of Science and Technology, Luoyang 471023, China; 00cyh00@163.com; 5 Department of Chemistry, Illinois State University, Normal, IL 61790-4160, USA

**Keywords:** SnO_2_, La_2_O_3_, heterojunction, ethanol, gas sensor, metal oxide semiconductor

## Abstract

Porous pure SnO_2_ nanofibers (NFs) and La_2_O_3_ nanoparticles (NPs)-embedded porous SnO_2_ NFs were successfully synthesized via electrospinning followed by calcination. These materials were systematically evaluated as gas-sensing elements in metal-oxide-semiconductor (MOS) sensors. The La_2_O_3_ NPs embedded in porous SnO_2_ NFs demonstrated superior gas-sensing performance compared to pure SnO_2_ NFs. Specifically, the incorporation of La_2_O_3_ resulted in a 12-fold enhancement in gas-sensing response towards ethanol, significantly improving both sensitivity and selectivity by tuning the carrier concentration and modifying oxygen deficiencies and chemisorbed oxygen levels. Thus, La_2_O_3_ NPs embedded in SnO_2_ NFs present a promising strategy for the development of high-performance ethanol gas sensors.

## 1. Introduction

Ethanol is a widely utilized volatile organic compound (VOC) with a broad spectrum of applications across various industries. Beyond its well-known role as an alternative fuel, ethanol is integral to sectors such as pharmaceuticals, chemical synthesis, and fermentation-based processes. Its significance is not only rooted in its extensive use but also in the need for precise monitoring of ethanol concentrations. Accurate detection is critical for ensuring product quality, maintaining safety standards, optimizing production efficiency, and complying with environmental regulations. As ethanol continues to play a pivotal role in both energy and industrial applications, the development of advanced sensing technologies for its effective monitoring has become increasingly important to fully harness its potential [1,2,3]. In the automotive industry, the use of ethanol as a biofuel is crucial for lowering greenhouse gas emissions and promoting sustainable energy. Accurate measurement of ethanol concentrations is vital for ensuring the quality of fuel blends and optimizing combustion efficiency. This precision is essential to advancing the global transition towards greener, more sustainable transportation solutions [4,5].

In pharmaceutical and chemical manufacturing, ethanol is a versatile solvent crucial for the extraction and synthesis of various compounds. Precise control of ethanol concentrations is essential to ensure the quality, consistency, and safety of pharmaceutical formulations and chemical processes. Accurate and selective ethanol gas sensors play a vital role in maintaining operational integrity, ensuring product standards, and minimizing potential hazards [6,7]. Detecting elevated alcohol levels in the breath of individuals who have consumed alcoholic beverages is vital for public safety, particularly in preventing drunk driving. Real-time monitoring of ethanol levels in both ambient air and a driver’s breath is essential to address this concern. Gas sensors that are simple to manufacture, easy to integrate, portable, and sufficiently sensitive are the preferred choice in this field. These advantages make them an effective solution for enhancing road safety by helping to monitor and prevent alcohol-impaired driving [8,9]. Given ethanol’s wide range of applications, the development of highly sensitive and selective gas sensors specifically for ethanol detection is essential. These sensors not only enhance safety and regulatory compliance but also improve process efficiency, reduce environmental impact, and support sustainable industrial practices.

MOSs have emerged as highly promising candidates for gas-sensing applications, owing to their exceptional sensitivity to alterations in the surrounding atmosphere [10,11]. Among these semiconductors, SnO_2_ and La_2_O_3_ have garnered substantial attention due to their distinctive properties. SnO_2_ is well-known as an n-type semiconductor, exhibiting remarkable gas-sensing properties, particularly in the detection of reducing gases [12]. Conversely, La_2_O_3_, recognized as a p-type semiconductor, stands out for its stability and distinctive electronic characteristics [13,14]. Recent research endeavors have delved into the synergistic advantages that arise from combining SnO_2_ and La_2_O_3_ in gas-sensing applications. A novel approach involves embedding La_2_O_3_ NPs within porous SnO_2_ NWs, aiming to augment the sensitivity and selectivity of ethanol gas sensors. The integration of SnO_2_ and La_2_O_3_ into a p-n junction establishes a platform that enhances the overall gas-sensing performance. SnO_2_ boasts several advantages, including a high surface area, excellent chemical stability, and sensitivity to a wide range of gases, making it an ideal candidate for the detection of ethanol vapors. Its capability to undergo significant changes in electrical conductivity in response to various gases further solidifies its suitability for gas-sensing applications [15,16]. Traditional ethanol sensors often face several key challenges that hinder their performance and practical applications. One of the primary issues is their requirement for high operating temperatures, typically above 300 °C, to achieve sufficient sensitivity. This not only increases energy consumption but also shortens the lifespan of the sensor, making it unsuitable for portable or low-power applications. Furthermore, conventional sensors often exhibit poor selectivity, struggling to distinguish ethanol from other VOCs such as acetone, methanol, and formaldehyde, which can lead to false readings or reduced accuracy in mixed gas environments. Additionally, many ethanol sensors have limited long-term stability due to factors such as material degradation, surface contamination, or drift in sensor response over time. This instability affects their reliability and necessitates frequent recalibration or replacement, which increases operational costs and limits their usability in long-term monitoring applications [17,18,19]. Complementarily, La_2_O_3_ introduces p-type conductivity to the semiconductor ensemble, contributing to the formation of a p-n junction. This particular junction structure enhances sensitivity and selectivity, as the presence of ethanol induces substantial alterations in the material’s electrical conductivity. The combined attributes of SnO_2_ and La_2_O_3_, integrated into a p-n junction, offer a robust foundation for achieving heightened performance in ethanol gas sensing, paving the way for advancements in gas sensing technology.

In this study, we synthesized La_2_O_3_ NPs-embedded porous SnO_2_ NFs using the electrospinning process, with a focus on investigating the effects of La_2_O_3_ doping on the ethanol-gas-sensing properties of SnO_2_ NFs. The incorporation of La_2_O_3_ as a dopant in the SnO_2_ matrix was systematically examined to understand its influence on key performance parameters, including sensitivity, detection limit, and response/recovery time. By carefully controlling the doping levels during the electrospinning process, we aimed to optimize the synergistic effects between La_2_O_3_ and SnO_2_, with the goal of significantly improving sensitivity, selectivity, and response/recovery times for ethanol detection. These improvements are essential for enhancing the practical application of ethanol sensors, particularly in environments requiring high sensitivity and fast response times at room temperature.

## 2. Materials and Methods

### 2.1. Materials

Tin(IV) chloride pentahydrate (SnCl_4_·5H_2_O, 98%), Lanthanum(III) nitrate hydrate (La(NO_3_)_3_·xH_2_O, 99.9%), and polyvinylpyrrolidone (PVP, Mw = 1,300,000) were sourced from Sigma-Aldrich (Saint Louis, MO, USA). N,N-Dimethylformamide (DMF) was supplied by DUKSAN PURE CHEMICALS (Ansan, Republic of Korea), while citric acid (C_6_H_8_O_7_, 99+%) was obtained from Alfa Aesar (Haverhill, MA, USA). Ethanol (99.5%) was purchased from Deajung Chemical (Siheung, Republic of Korea). Various gases (99.99%) used for sensing performance measurements were provided by Samjung Special Gas (Incheon, Republic of Korea). All other chemicals and reagents were used as received, without further purification unless specified otherwise.

### 2.2. Preparation

**Figure 1:**
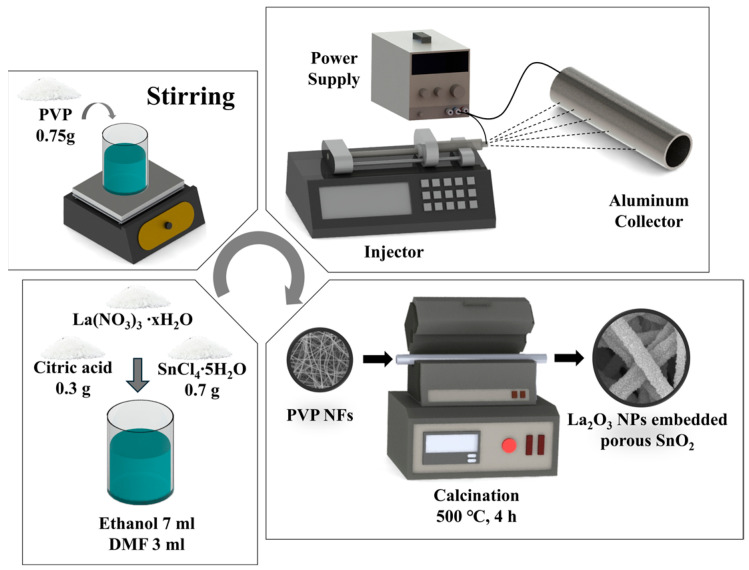
Schematic diagrams of the synthesis of La_2_O_3_ NPs-embedded porous SnO_2_ NFs.

Pure porous SnO_2_ NFs and La_2_O_3_ NPs-embedded porous SnO_2_ NFs with varying La_2_O_3_ concentrations were synthesized through a meticulous electrospinning process, followed by calcination. To begin, a solution was prepared by dissolving 0.3 g of citric acid, 0.7 g of SnCl_4_·5H_2_O, and specific amounts of La(NO_3_)_3_·xH_2_O (0, 0.016, 0.032, and 0.048 g) in a solvent mixture of 7 mL absolute ethanol and 3 mL DMF. This mixture was stirred magnetically at room temperature for 30 min. Next, 0.75 g of PVP was gradually added, followed by an additional 30 min of stirring at room temperature. The prepared solution was loaded into a 7 mL plastic syringe for electrospinning, as shown in Figure 1. A DC voltage of 12 kV was applied between the syringe needle and a collector positioned 20 cm apart, with a feed rate maintained at 0.005 mL/min. The resulting white fibers were collected and calcined in a tube furnace at 500 °C for 4 h. The samples were labeled 0La/SnO_2_, 16La/SnO_2_, 32La/SnO_2_, and 48La/SnO_2_, corresponding to the respective La(NO_3_)_3_·xH_2_O ratios (0, 0.016, 0.032, and 0.048 g).

### 2.3. Characterization

The morphologies of the samples were carefully analyzed using field emission-scanning electron microscopy (FE-SEM, Hitachi S8010, HITACHI, Tokyo, Japan). The crystal structures were characterized by X-ray diffraction (XRD) patterns obtained at a 5° glancing angle with a high-resolution X-ray diffractometer (D/Max-2500/PC, Rigaku, Tokyo, Japan) using CuKα radiation (λ = 1.5418 Å) over a scattering angle range of 20° to 80°. For more detailed analysis of the samples’ morphology and crystal structure, field-emission transmission electron microscopy (FE-TEM, Jeol 2100F, JEOL, Tokyo, Japan) equipped with energy-dispersive X-ray spectroscopy (EDS) was employed for qualitative chemical composition analysis of the nanofibers. X-ray photoelectron spectroscopy (XPS, PHI 5000 Versa Probe II, ULVAC-PHI, Chigasaki, Japan) was used to accurately determine the binding energies of the samples. Additionally, the specific surface area and pore size distribution were measured through N_2_ gas adsorption using Brunauer–Emmett–Teller (BET, BELSORP-max, MicrotracBEL Corp., Osaka, Japan) and Barrett–Joyner–Halenda (BJH) surface analysis techniques.

### 2.4. Fabrication and Measurement of Gas Sensor

In this study, a gas sensor device was used to assess gas-sensing performance within a dedicated system, as shown in Figure S1. Interdigitated Electrode (IDE) chips were fabricated through a lift-off process, involving the sequential deposition of a Ti adhesion layer and an Au conduction layer on a SiO_2_/Si wafer, with a 10 μm gap between electrodes. The material was dispersed in ethanol and sonicated, after which the suspension was applied to the IDE chip and air-dried. This process was repeated three times to ensure sufficient sample deposition on the chip. Gas-sensing performance was evaluated using a custom-designed system featuring a quartz tube as the sensing chamber. The IDE chip was placed at the center of the chamber and connected to a source-meter unit (SMU, Keithley 2450). Before testing, the gas was dried to 0.7% humidity using a DRIERITE gas drying unit (W.A. Hammond Drierite Co. Ltd., Xenia, OH, USA). The response performance of the target gas was tested under varying relative humidity (RH) conditions; the RH of the air was set to 0%, 20%, 40%, and 80%, with specific humidity control referenced in Figure S2. During the experiments, the gas concentration was adjusted to five levels: 0.5, 1, 2, 5, 10, and 20 ppm. The controlled gas injection rate of 20 sccm maintained stable pressure conditions, effectively minimizing the impact of pressure fluctuations on resistance measurements. After each introduction of the target gas, synthetic air was used as the purging gas and systematically delivered to the IDE chips. The target gas and purging gas were supplied to the sensors for 200 s and 1000 s, respectively. Resistance data from the sensors were recorded by the source-meter at one-second intervals throughout the process. Sensor response was defined as R_a_/R_g_, where R_a_ is the sensor resistance in air and R_g_ in the target gas [20]. Response and recovery times were measured when the resistance reached 90% of its total shift during adsorption and desorption.

## 3. Results

### 3.1. Characterization of La_2_O_3_ NPs-Embedded Porous SnO_2_ NFs

**Figure 2:**
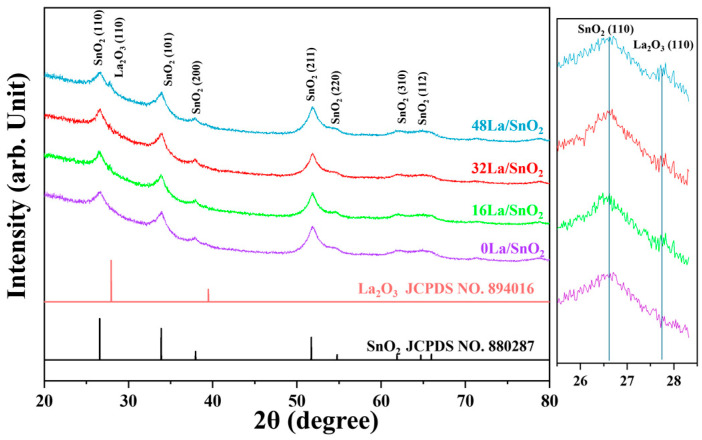
XRD patterns of the samples synthesized in this study.

Figure 2 shows the XRD patterns for 0La/SnO_2_, 16La/SnO_2_, 32La/SnO_2_, and 48La/SnO_2_, representing the composites of SnO_2_ and La_2_O_3_. The XRD pattern of 0La/SnO_2_ corresponds to the tetragonal structure of SnO_2_ (JCPDS No. 88–0287), with no additional peaks, confirming the purity of the SnO_2_ NFs. In the other samples, the XRD patterns exhibit additional peaks attributed to the body-centered cubic structure of La_2_O_3_ (JCPDS No. 89–4016), indicating the successful incorporation of La_2_O_3_. However, due to the relatively low concentration of La_2_O_3_ compared to SnO_2_, most characteristic La_2_O_3_ peaks are not prominent, and only the (110) peak of La_2_O_3_ is observed in the samples. The intensity of these La_2_O_3_ peaks increases proportionally with the concentration of La_2_O_3_ NPs, while the SnO_2_ peaks remain nearly constant across all samples, confirming the structural stability of SnO_2_. Notably, no peaks corresponding to any secondary phases or impurities were observed. A high-resolution XRD figure has been provided to clearly illustrate these peak characteristics.

**Figure 3:**
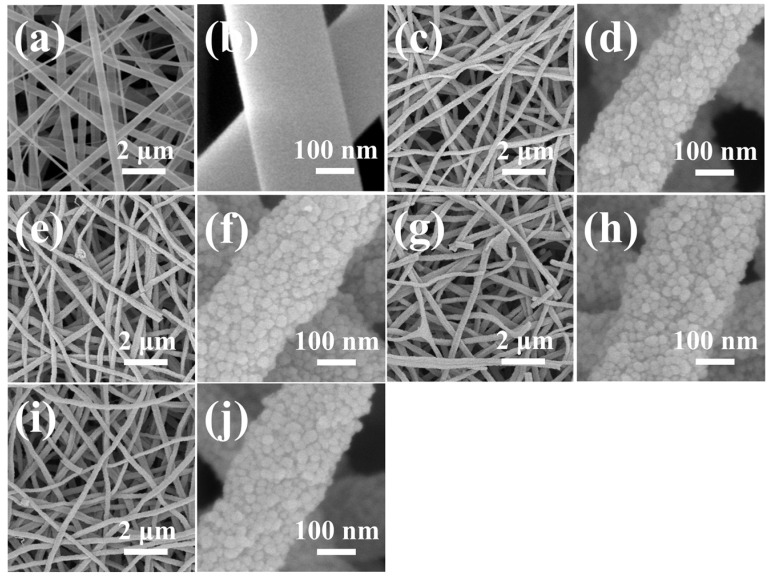
Low-magnification SEM images of (**a**) PVP NFs, (**c**) 0La/SnO_2_, (**e**) 16La/SnO_2_, (**g**) 32La/SnO_2_, and (**i**) 48La/SnO_2_. (**b**–**j**) show the corresponding high-magnification SEM images.

The NF structure underwent significant changes due to thermal calcination, which removed the PVP and transformed the embedded salts within the polymer matrix into metal oxide grains through oxidation and crystallization. SEM images with detailed magnifications of the synthesized samples are shown in Figure 3. A low-magnification SEM image of the PVP NFs before calcination is presented in Figure 3a, while post-calcination images for 0La/SnO_2_, 16La/SnO_2_, 32La/SnO_2_, and 48La/SnO_2_ are displayed in Figure 3c, Figure 3e, Figure 3g, and Figure 3i, respectively. Corresponding high-magnification images are provided in Figure 3b,d,f,h,j. These images indicate that the smooth NFs, initially around 300 nm in diameter, transitioned into porous, uneven structures composed of small sub-grains after calcination. These sub-grains, containing both SnO_2_ and La_2_O_3_, were difficult to distinguish due to their similar appearances. After calcination, the NF diameter reduced to approximately 250 nm, primarily due to the removal of the polymer matrix. Despite varying La_2_O_3_ NPs concentrations, the morphologies of the calcined NFs remained largely consistent across all samples.

**Figure 4:**
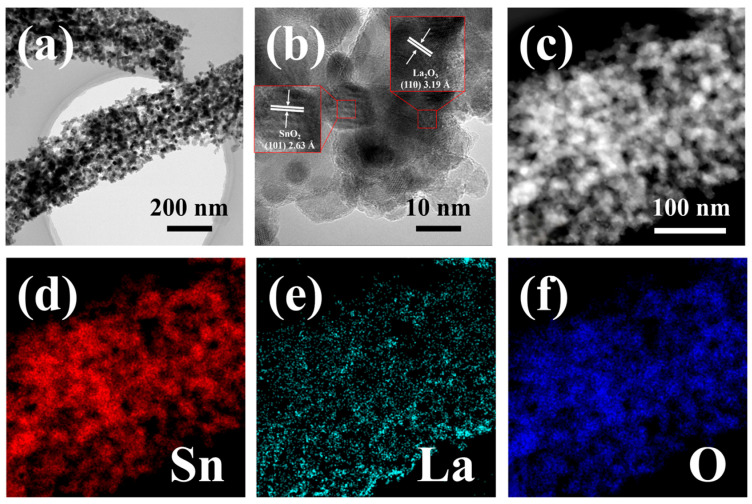
TEM images of 32La/SnO_2_ include (**a**) a low-magnification image displaying the complete morphology of the sample; (**b**) a high-resolution, enlarged image revealing individual sub-grains, including SnO_2_ and La_2_O_3_, along with fringe patterns that highlight the lattice planes; (**c**) a STEM image of the NF; and EDS elemental mapping profiles derived from the STEM image showcasing the distribution of (**d**) Sn, (**e**) La, and (**f**) O.

Figure 4a–f present the TEM images and corresponding EDS elemental mapping profiles for 32La/SnO_2_. In the low-magnification image (Figure 4a), the overall morphology of the NF is visible, consistent with the SEM analysis, showing numerous sub-grains and a porous structure. The high-resolution TEM image in Figure 4b reveals sub-grains varying in size from 5 to 30 nm, with clear fringe patterns visible within the grains. Two distinct fringe spacings, 2.63 Å and 3.19 Å, correspond to the (101) lattice plane of SnO_2_ and the (110) lattice plane of La_2_O_3_, respectively, indicating interconnected nanoparticles across grain boundaries. The STEM image of the NF is shown in Figure 4c, while Figure 4d–f provide the EDS elemental mapping profiles for Sn, La, and O based on the STEM image. These profiles demonstrate a uniform distribution of elements throughout the sample, indicating that La_2_O_3_ nanoparticles are evenly dispersed within the SnO_2_ NFs, highlighting their high dispersibility.

**Figure 5:**
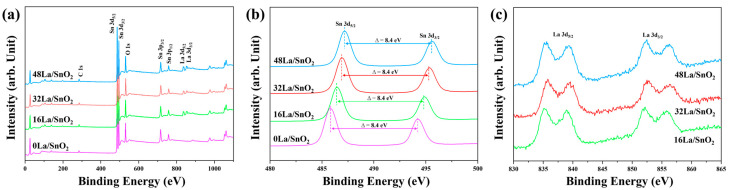
(**a**) The fully scanned survey spectra of the 0La/SnO_2_, 16La/SnO_2_, 32La/SnO_2_, and 48La/SnO_2_, along with their corresponding high-resolution XPS spectra analysis (**b**) Sn 3d, and (**c**) La 3d.

The surface chemical composition and chemical states of the NFs were elucidated using XPS, as depicted in Figure 5. The fully scanned survey spectra for 0La/SnO_2_, 16La/SnO_2_, 32La/SnO_2_, and 48La/SnO_2_ are showcased in Figure 5a. These spectra utilized the C 1s peak at a binding energy of 284.6 eV for calibration [21]. In all samples, distinct peaks corresponding to Sn 3d and O 1s were observed. Furthermore, La 3d peaks appeared in the 16La/SnO_2_, 32La/SnO_2_, and 48La/SnO_2_, affirming the incorporation of lanthanum. The absence of La 3d peaks in 0La/SnO_2_ indicates a lack of lanthanum in this sample. Figure 5b highlights the Sn 3d peaks, which are prominently positioned at approximately 486 eV and 494 eV, corresponding to the Sn 3d_5/2_ and Sn 3d_3/2_ energy levels, respectively, with a consistent energy separation of 8.4 eV between them. This separation is consistent with the values associated with Sn^4+^ in SnO_2_, confirming the complete oxidation state of Sn^4+^ in these samples, as reported in the literature [22,23]. The slight shift in peak positions can be attributed to the electronic interaction between SnO_2_ and La_2_O_3_, which significantly influences the local chemical environment and binding energies. The difference in work functions between these two materials induces electron transfer at the interface, thereby modifying the electronic structure and resulting in the observed shifts in the XPS spectra. This electronic interaction further confirms the successful incorporation of La_2_O_3_ into the composite and supports the formation of a heterojunction between SnO_2_ and La_2_O_3_. The heterojunction not only alters the electronic structure but also enhances charge carrier separation at the interface, reducing recombination rates. Moreover, the potential barrier created by the heterojunction affects the mobility of charge carriers, which is critical for improving the sensing performance of the composite. This enhanced charge separation and mobility contribute directly to the superior gas-sensing properties of the material [24,25]. In Figure 5c, the high-resolution XPS spectra for La 3d in the 16La/SnO_2_, 32La/SnO_2_, and 48La/SnO_2_ reveal peaks corresponding to La 3d_5/2_ at 836 eV and La 3d_3/2_ at 853 eV, with a consistent energy gap of 16.78 eV between these levels. This gap substantiates the presence of the La^3+^ oxidation state [26,27,28].

**Figure 6:**
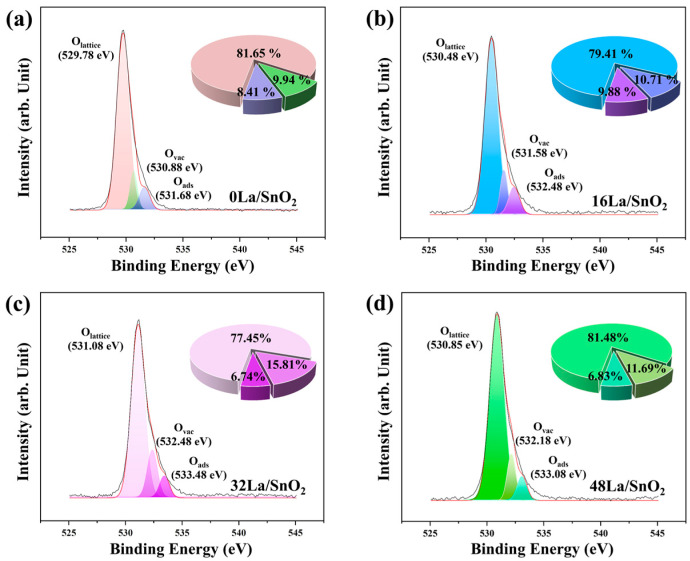
The high-resolution XPS spectra of (**a**) 0La/SnO_2_, (**b**) 16La/SnO_2_, (**c**) 32La/SnO_2_, and (**d**) 48La/SnO_2_ for the O 1s, along with the quantified proportions of O_lattice_, O_ads_, and O_vac_ within the O 1s.

Figure 6 presents the broad and asymmetric spectra of O 1s for the series of samples: 0La/SnO_2_, 16La/SnO_2_, 32La/SnO_2_, and 48La/SnO_2_. The O 1s peak is differentiated into three distinct components: lattice oxygen (O_lattice_), oxygen vacancies (O_vac_), and chemisorbed oxygen species (O_ads_) [29]. The O_lattice_ component, representing oxygen ions within the metal ion framework, exhibits remarkable stability and shows no reactivity towards reducing gases [30]. The O_vac_ component, associated with dissociative oxygen species, offers active sites for gas adsorption and catalytic reactions on the surface of the sensing materials, indicating that an increase in O_vac_ enhances the participation of chemisorbed oxygen in surface oxidation-reduction reactions, thereby amplifying the gas-sensing response. This component is particularly crucial due to its role in interacting with the target gas [31]. O_ads_, often linked to chemisorbed oxygen species, is directly involved in the surface redox reactions with target molecules on the sensitive materials [32]. Figure 6 details the relative proportions and distributions of O_lattice_, O_vac_, and O_ads_ across the samples, highlighting that the ratio of (O_vac_ + O_ads_) in 32La/SnO_2_ is superior, rendering it more effective for gas-sensing purposes. The rise in O_vac_ is attributed to the generation of oxygen vacancies and deficiency regions, which are induced by the incorporation of La_2_O_3_ into SnO_2_, thereby enhancing gas-sensing capabilities [33,34].

**Figure 7:**
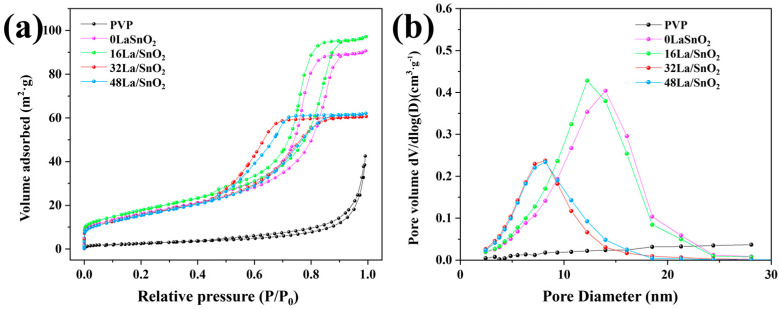
(**a**) The N_2_ adsorption–desorption isotherms for PVP NFs embedded with tin and lanthanum salts, 0La/SnO_2_, 16La/SnO_2_, 32La/SnO_2_, and 48La/SnO_2_, accompanied by their respective (**b**) pore size distributions.

Figure 7a presents the nitrogen adsorption and desorption isotherms for PVP NFs and a series of La-doped SnO_2_ samples, including 0La/SnO_2_, 16La/SnO_2_, 32La/SnO_2_, and 48La/SnO_2_. Based on BET analysis, all samples, except for PVP NFs, exhibit type IV nitrogen adsorption–desorption isotherms with H2 hysteresis loops, characteristic of a mesoporous structure. The specific surface areas, calculated using the BET method, were found to be 9.83 m^2^/g for PVP NFs, and 59.23, 65.25, 58.41, and 57.32 m^2^/g for 0La/SnO_2_, 16La/SnO_2_, 32La/SnO_2_, and 48La/SnO_2_, respectively. Notably, following calcination, the surface areas of the SnO_2_-based samples increased more than sixfold compared to the uncalcined PVP NFs. This significant rise in surface area can be attributed to the formation of pores during calcination, which enhances the surface reactivity by providing numerous active sites for chemical interactions. The increase in active sites significantly boosts the sensor’s performance by enabling more efficient interactions with target gases, thereby improving both sensitivity and specificity. Additionally, pore size distribution, determined from the desorption branch using the Barrett–Joyner–Halenda (BJH) method (as depicted in Figure 7b), revealed an almost negligible average pore size for the nonporous PVP NFs. In contrast, the pore sizes for 0La/SnO_2_, 16La/SnO_2_, 32La/SnO_2_, and 48La/SnO_2_ were calculated to be 9.45, 9.20, 6.42, and 6.69 nm, respectively. These results from the BET and BJH analyses are consistent with the observations from SEM and TEM imaging, further confirming the porous structure of the calcined nanofibers. The enhanced sensing performance of the calcined samples can be primarily attributed to the increased volume of mesopores, which facilitates better gas diffusion and interaction.

### 3.2. Gas-Sensing Properties

**Figure 8:**
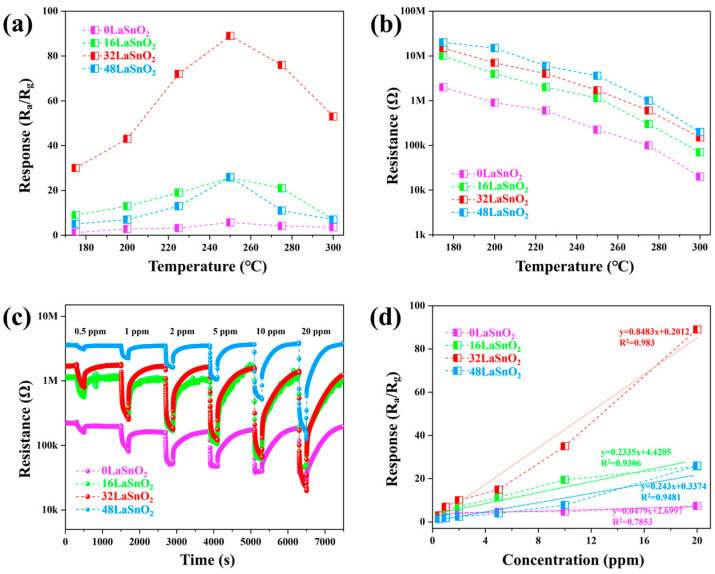
For sensors based on 0La/SnO_2_, 16La/SnO_2_, 32La/SnO_2_, and 48La/SnO_2_: (**a**) response curves to 20 ppm ethanol as a function of operating temperature; (**b**) baseline resistance changes as a function of operating temperature; (**c**) dynamic response transients at 250 °C as a function of ethanol concentration; (**d**) fitted response curves at 250 °C as a function of ethanol concentration.

In this study, ethanol-sensing tests were conducted on La_2_O_3_/SnO_2_ composite materials to evaluate their optimal operating temperature, sensitivity, linear dependence on ethanol concentration, and selectivity. Operating temperature plays a critical role in gas sensor performance, as it significantly affects the surface reactions during gas sensing. To identify the optimal temperature, the responses of the four sensor samples developed in this study were analyzed across a temperature range of 175–300 °C, using 20 ppm ethanol as the target gas. As shown in Figure 8a, the gas response varies with temperature for all samples, exhibiting a typical rise and fall pattern. At lower temperatures, ethanol molecules have difficulty reacting effectively with the oxygen adsorbed on the sensor surface, leading to a reduced sensor response. As the temperature increases, the reaction rate accelerates, enhancing the conversion of surface-adsorbed oxygen and improving the sensor’s response. However, as the temperature rises beyond the optimal point, the sensor response declines due to several factors. Firstly, ethanol molecules desorb more quickly at higher temperatures, reducing their interaction with surface-adsorbed oxygen and limiting the release of electrons back into the conduction band. Secondly, elevated temperatures decrease oxygen adsorption, thinning the depletion layer and diminishing the sensor’s sensitivity. Lastly, at very high temperatures, the rate of surface reactions can reach a saturation point where further increases in temperature no longer enhance the reaction kinetics. The surface reaction between ethanol and oxygen becomes less efficient as the temperature increases, leading to a lower number of available electrons to be released back into the conduction band, thus reducing the sensor’s response. An operating temperature of 250 °C was identified as optimal, balancing the benefits of a higher reaction rate with sufficient adsorption capacity. Figure 8b shows the variation in baseline resistance of the sensors over the operating temperature range of 175 to 300 °C. The baseline resistance of 0La/SnO_2_, 16La/SnO_2_, 32La/SnO_2_, and 48La/SnO_2_ sensors followed a similar trend with increasing temperature. As temperature rises, the concentration of charge carriers (electrons or holes) in the material increases due to thermal excitation, leading to more electron-hole pair generation. In metal oxide semiconductors, this results in higher conductivity and consequently lower resistance [35,36]. Figure 8c illustrates the dynamic response curves for the four sensors at various ethanol concentrations, recorded at an operating temperature of 250 °C. All sensors exhibit behavior characteristic of n-type semiconductor-based gas sensors. Across ethanol concentrations ranging from 1 to 20 ppm, the sensors demonstrated optimal response and recovery characteristics, indicating excellent reversibility and sensor stability. Figure 8d presents the sensor responses as a function of ethanol concentration at 250 °C. Notably, the response increases proportionally with ethanol concentration for all sensors within the 1 to 20 ppm range. Among them, the 32La/SnO_2_ sensor exhibited a significantly higher response than the others, with responses to 20 ppm ethanol at 250 °C measured at 7.4, 25.7, 89, and 26 for the 0La/SnO_2_, 16La/SnO_2_, 32La/SnO_2_, and 48La/SnO_2_ sensors, respectively. The 32La/SnO_2_ sensor showed a response approximately 12 times greater than that of 0La/SnO_2_, indicating its superior detection performance with high linearity for ethanol concentrations as low as 1 ppm, without the need for noble metal catalysts such as Au, Pd, or Pt. Furthermore, the sensitivity of the 0La/SnO_2_, 16La/SnO_2_, 32La/SnO_2_, and 48La/SnO_2_ sensors to ethanol concentration was analyzed using linear fitting, resulting in the following equations: y = 0.0479x + 2.6997 (R^2^ = 0.7853), y = 0.2335x + 4.4205 (R^2^ = 0.9306), y = 0.8483x + 0.2012 (R^2^ = 0.983), and y = 0.243x + 0.3374 (R^2^ = 0.9481), respectively. The coefficient of determination (R^2^) for the 32La/SnO_2_ sensor was close to 1.0, indicating excellent linearity. The standard deviation (S) was calculated using the root-mean-square deviation (RMS) formula: RMSnoise = (S^2^/N)^1/2^, where N represents the total number of data points. Based on the formula DL = 3.3 (RMSnoise/slope), the limit of detection (LOD) for the 32La/SnO_2_ sensor was determined to be 58 ppb, significantly lower than 0.5 ppm, underscoring its potential for detecting low concentrations of ethanol.

**Figure 9:**
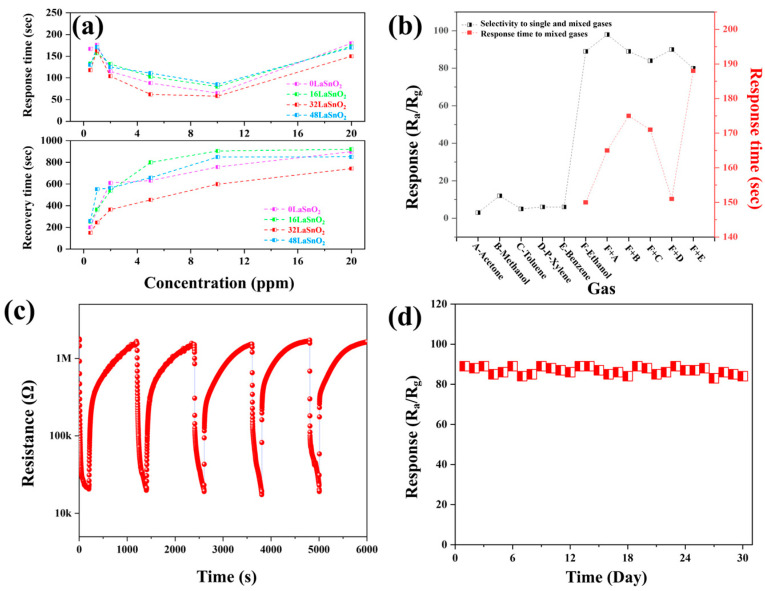
(**a**) Response and recovery time curves of 0La/SnO_2_, 16La/SnO_2_, 32La/SnO_2_, and 48La/SnO_2_-based sensors at 250 °C as a function of ethanol concentration; (**b**) selectivity to single and mixed gases and response time to mixed gases of the 32La/SnO_2_-based sensor to 20 ppm ethanol and various gas mixtures at 250 °C; (**c**) reversibility of the 32La/SnO_2_-based sensor upon exposure to 20 ppm ethanol at 250 °C; (**d**) daily response values of the 32La/SnO_2_-based sensor to 20 ppm ethanol over 30 days at 250 °C.

The response and recovery time curves of the 0La/SnO_2_, 16La/SnO_2_, 32La/SnO_2_, and 48La/SnO_2_ sensors at different ethanol concentrations, recorded at 250 °C, are presented in Figure 9a. As the ethanol concentration increased from 1 to 20 ppm, the 32La/SnO_2_-based sensor demonstrated superior performance compared to the other three sensors. Figure 9b explores the selectivity of the gas sensors, a critical parameter for assessing their quality in real-world applications. The responses of all sensors to 20 ppm of various volatile organic compounds (VOCs), including ethanol, acetone, methanol, toluene, p-xylene, and benzene, were measured. Notably, the 32La/SnO_2_ sensor exhibited the highest selectivity for ethanol among the tested gases. Additionally, the selectivity of the 32La/SnO_2_ sensor for ethanol in mixed gas environments was evaluated at the optimal operating temperature of 250 °C. For binary gas mixtures, the responses of the 32La/SnO_2_ sensor to gases containing ethanol were close to the original ethanol response (98, 89, 84, 90, and 80), demonstrating that the sensor maintained high selectivity for ethanol even in the presence of other gases. This indicates the sensor’s effectiveness in detecting ethanol amidst complex gas mixtures. During the response time tests for binary gas mixtures, although the response times were generally higher than for single ethanol gas, the overall difference was not significant. This indicates that the sensor maintains excellent selectivity for ethanol even in the presence of other gases. As shown in Figure 6c, the long-term stability of the 32La/SnO_2_ sensor was tested with 20 ppm ethanol at 250 °C over a 30-day period. The sensor’s gas response remained stable around 89, with fluctuations within 5%, which is well within the acceptable range. This suggests that the 32La/SnO_2_ sensor exhibits outstanding long-term stability, making it well-suited for practical applications. Finally, Figure 9d shows the results of subjecting the 32La/SnO_2_ sensor to five cycles of exposure to 20 ppm ethanol. The sensor displayed consistent response and recovery characteristics across all cycles, highlighting its excellent repeatability and robustness in gas-sensing applications.

**Figure 10:**
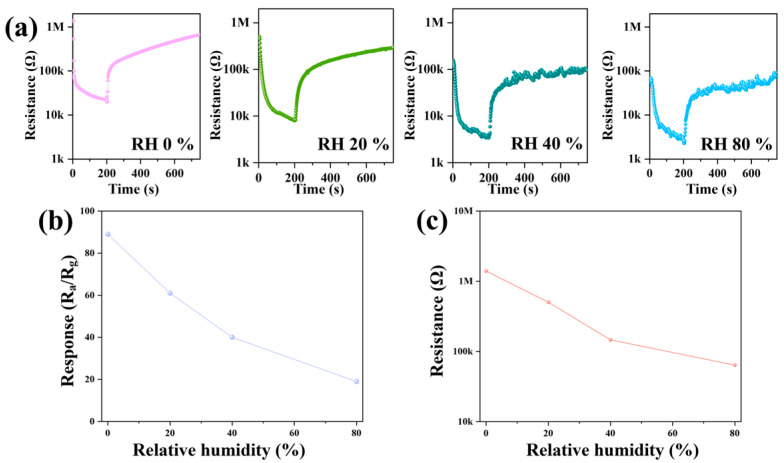
(**a**) Dynamic response curves of 32La/SnO_2_-based sensors to 20 ppm ethanol across different levels of RH, along with the corresponding (**b**) response and (**c**) baseline resistance relationships.

For semiconductor oxide gas sensors, sensing performance is significantly affected by RH. To investigate this, the response of the 32La/SnO_2_-based sensor to 20 ppm ethanol under varying RH levels was studied, and the results are shown in Figure 10. The dynamic response curves in Figure 10a demonstrate that both the response of the 32La/SnO_2_ sensor to 20 ppm ethanol and its baseline resistance decrease as humidity increases. Figure 10b,c further illustrate that as RH rises from 0% to 80%, both the sensor’s response and baseline resistance decrease in an approximately linear fashion. The reduction in the sensor’s performance can be attributed to the diminished availability of adsorption sites on the material’s surface, as water vapor competes with ethanol molecules for oxygen species. Additionally, the observed decline in baseline resistance with increasing humidity is likely due to the accumulation of water molecules on the sensor’s surface, which enhances conductivity and reduces resistance [37,38,39].

Comparison and analysis using Table 1 of the reported ethanol gas-sensing characteristics indicate that our device exhibits higher response values. This suggests significant prospects for our device in ethanol gas detection.

### 3.3. Gas-Sensing Mechanism

**Figure 11:**
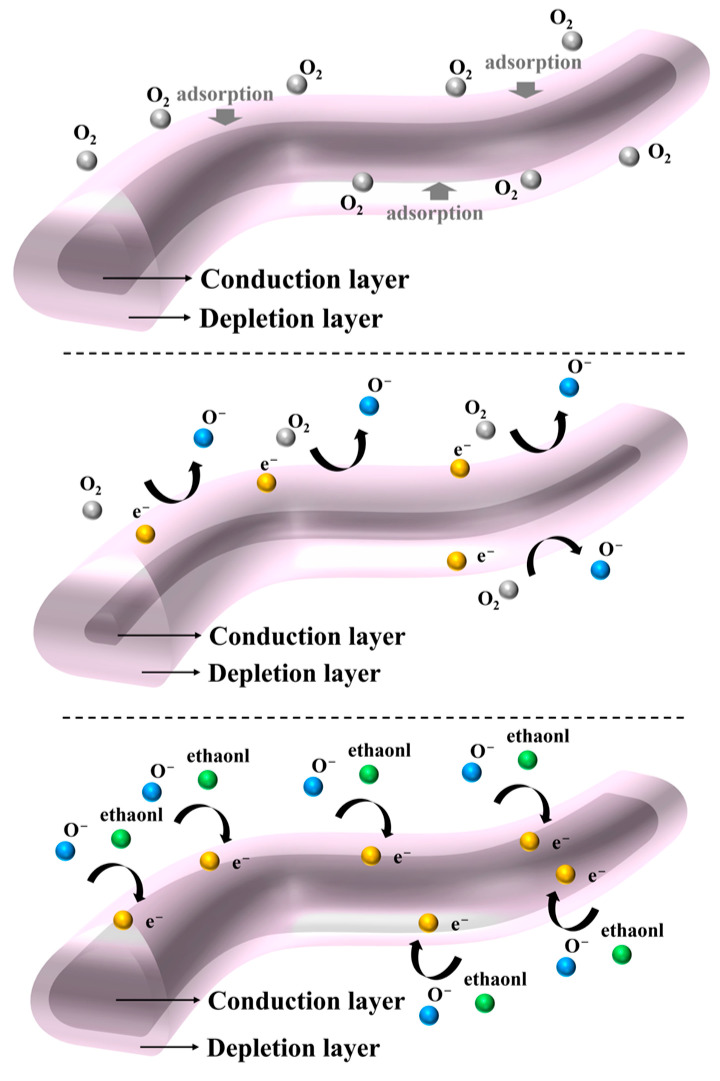
Schematic diagrams illustrating the electrical structure of NFs when exposed to air and ethanol gas.

The chemisorption of analyte gases causes significant changes in the electrical properties, particularly the resistance, of MOS-based gas sensors [50]. SnO_2_, being an n-type semiconductor, and La_2_O_3_, a p-type semiconductor metal oxide, form p-n heterojunctions when La_2_O_3_ is incorporated into the SnO_2_ matrix. This leads to the migration of electrons in SnO_2_ and holes in La_2_O_3_ in opposite directions as they seek equilibrium at similar Fermi energy levels [51]. As observed in Figure 8c, the La_2_O_3_/SnO_2_-based sensor exhibits an n-type response, suggesting that the sensitivity is primarily governed by changes in electron concentration in SnO_2_. The ethanol-sensing mechanism, based on the surface space charge layer model, can be explained more thoroughly through three distinct steps, as illustrated in Figure 11. First, in the presence of air, oxygen molecules from the atmosphere adsorb onto the active sites on the surface of the sensing material. These oxygen molecules then dissociate and capture electrons from the conduction band of the semiconductor, forming oxygen anion species such as O_2_⁻, O⁻, and O^2^⁻. This process creates a depletion layer near the surface, where the electron concentration is reduced, leading to an increase in the sensor’s resistance. The adsorption of oxygen is a dynamic and reversible process, where the continuous adsorption and desorption of oxygen molecules occur until a steady state is reached, which stabilizes the sensor’s baseline resistance (Equations (1)–(4)) [52]. When the sensor is exposed to ethanol, the ethanol molecules interact with the surface-adsorbed oxygen anions. Ethanol undergoes an oxidation reaction with the oxygen species, converting to CO_2_ and H_2_O, and releasing the trapped electrons back into the conduction band of the semiconductor. This process reduces the width of the depletion layer and causes a decrease in the sensor’s resistance. The change in resistance is directly related to the concentration of ethanol in the surrounding environment. However, this reaction is opposed by the simultaneous re-adsorption of oxygen from the air, which competes for the same active sites and tends to re-establish the depletion layer, moderating the sensor’s overall response to ethanol (Equation (5)) [47]. Once the ethanol is removed from the environment, oxygen from the air re-adsorbs onto the surface of the sensor, following the same mechanism as in the first step. The oxygen molecules again capture electrons from the conduction band, reforming the depletion layer and returning the sensor’s resistance to its original state. This recovery phase is essential for demonstrating the sensor’s ability to maintain repeatability and long-term stability over multiple cycles of exposure to ethanol and subsequent recovery.

(1) $$O_{2}\;(gas)\to O_{2}\;(ads)$$

(2) $$O_{2}\;(ads)+e^{-}\to O_{2}^{-}\;(ads)$$

(3) $$O_{2}^{-}\;(ads)+e^{-}\to {2O}^{-}\;(ads)$$

(4) $$O^{-}\;(ads)+e^{-}\;\to O^{2-}\;(ads)$$

(5) $$C_{2}H_{5}OH\;(gas)+6O^{-}\;(ads)\to 2{CO}_{2}\;(gas)+{3H}_{2}O\;(gas)+{6e}^{-}$$

**Figure 12:**
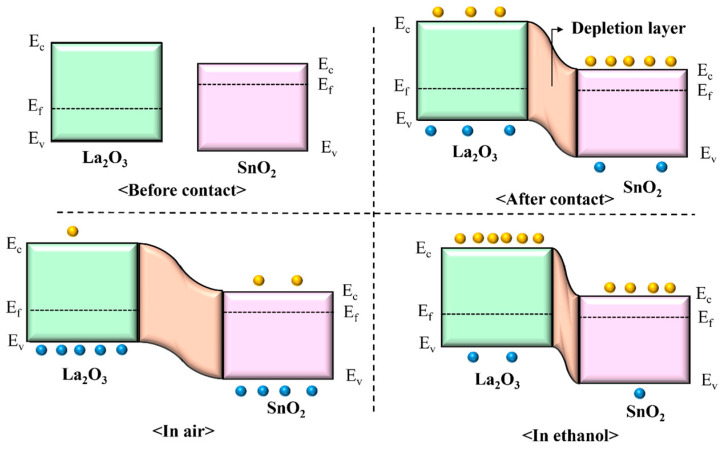
Schematic diagram illustrating the gas-sensing mechanism and energy band structure of La_2_O_3_/SnO_2_.

The La_2_O_3_/SnO_2_ composites exhibit a significantly enhanced response to ethanol sensing, primarily due to the formation of a p-n junction at the interface between SnO_2_ and La_2_O_3_. This interface introduces several beneficial effects. The ethanol-sensing mechanism for this heterojunction sensor is schematically illustrated in Figure 12. Initially, the band diagrams of SnO_2_ and La_2_O_3_ show their majority carriers—electrons for SnO_2_ and holes for La_2_O_3_—before the formation of the interface [53]. Upon the formation of the interface between SnO_2_ and La_2_O_3_, the difference in their energy band structures leads to band bending at the junction. This band bending facilitates the migration of charge carriers—specifically, electrons and holes—across the heterojunction, contributing to the formation of a depletion region at the interface. The depletion region is characterized by a lower carrier concentration and the establishment of a potential barrier, which impedes the free movement of charge carriers. Consequently, the reduced carrier concentration in this region, coupled with the potential barrier, significantly increases the resistance of the composite material when compared to pure SnO_2_. The presence of La_2_O_3_ further enhances this effect due to its catalytic properties and its role in increasing oxygen vacancies in the SnO_2_ matrix, which improves the adsorption of oxygen species on the surface. These oxygen species capture free electrons from SnO_2_, further expanding the depletion region and elevating the potential barrier. As a result, the overall electric current is reduced, leading to an increase in resistance. This combination of effects explains the enhanced sensing performance, as the presence of La_2_O_3_ improves the material’s response to changes in gas concentration by modulating the carrier dynamics and the depletion region at the heterojunction. This is confirmed by the data in Figure 8c, where the resistance of the La_2_O_3_/SnO_2_ composite ranges from 1.29 to 3.82 MΩ, significantly higher than the 0.188 MΩ observed for pure SnO_2_. When ethanol is introduced into the sensing chamber, it interacts with the chemisorbed oxygen species on the sensor’s surface, as described in Equation (5). This reaction leads to the oxidation of ethanol, releasing electrons back into the conduction band of the sensing material, which increases the carrier concentration and consequently decreases the sensor’s resistance. The presence of La_2_O_3_ in the SnO_2_ matrix significantly enhances this process by creating a p-n heterojunction at the interface. The difference in lattice spacing between SnO_2_ (n-type) and La_2_O_3_ (p-type) at the p-n junction introduces defects and vacancies near the interface, which serve as additional active sites for gas adsorption and surface reactions. This increased density of active sites allows for more ethanol molecules to participate in the surface reaction, leading to a more pronounced reduction in the depletion layer’s width and a sharper decrease in resistance. Moreover, the heterojunction at the SnO_2_/La_2_O_3_ interface creates a potential barrier that modulates the flow of charge carriers, further enhancing the sensitivity of the sensor. The defects and vacancies near the junction not only improve gas adsorption but also facilitate faster electron transfer during the ethanol oxidation process. As a result, the resistance change in the La_2_O_3_/SnO_2_ composite is significantly larger than in pure SnO_2_, highlighting the critical role of the heterojunction in amplifying the sensor’s response to ethanol. This enhanced sensitivity can be attributed to the synergistic effects of the p-n heterojunction, increased active sites for gas adsorption, and the improved electron mobility in the composite material. These factors together make the La_2_O_3_/SnO_2_ composite highly effective for ethanol detection, offering superior sensitivity and faster response times compared to undoped SnO_2_.

## 4. Conclusions

In this study, La_2_O_3_ NPs were successfully incorporated into porous SnO_2_ NFs using a simple two-step process of electrospinning followed by calcination. The SnO_2_ NFs doped with an optimal amount of La_2_O_3_ NPs (32La/SnO_2_) demonstrated a significantly enhanced response to 20 ppm ethanol, achieving a response value of 89—12 times higher than that of undoped SnO_2_ NFs. This substantial improvement in ethanol detection can be attributed to the combined effects of the porous structure, which provides a large specific surface area, and the formation of p-n heterojunctions between La_2_O_3_ and SnO_2_. The increased surface area offers a greater number of active sites for gas adsorption, maximizing the utilization of the sensing material. Furthermore, the p-n heterojunctions formed at the La_2_O_3_/SnO_2_ interfaces enhance electron transport, further boosting the material’s gas-sensing capability. The synergistic effects of the high surface area and efficient electron transfer through the heterojunctions collectively contribute to the significant enhancement in gas-sensing performance observed in the La_2_O_3_/SnO_2_ composite.

## Figures and Tables

**Table 1:** Comparative analysis of ethanol-sensing performance among various oxide-based gas sensors.

Sensing Materials	Con. (ppm)	Tem. (°C)	Res. (R_a_/R_g_)	Res. Time (s)	Rec. Time (s)	Ref.
Cr_2_O_3_/ZnS	200	300	13.84	23	20	[40]
In_2_O_3_/ZnO	100	225	32	3.7	52	[41]
WO_3_	200	300	32.5	6	8	[42]
In_2_O_3_/ZnS	100	300	15	40	225	[43]
In_2_O_3_	100	280	45	-	-	[44]
La_2_O_3_/SnO_2_	100	400	57.3	-	-	[45]
LaFeO_3_	143	300	14.5	23	39	[46]
NiO/LaFeO_3_	10	240	14.7	2	9	[47]
Au/La/In_2_O_3_	100	210	1.48	1	394	[48]
Au@Cr_2_O_3_-In_2_O_3_	5	180	4.4	135	618	[49]
La_2_O_3_/SnO_2_	20	250	111	150	742	This work

## Data Availability

Data are contained within the article.

## Supplementary Materials

The following supporting information can be downloaded at https://www.mdpi.com/article/10.3390/s24216839/s1, Figure S1: Schematic diagram of homemade gas-sensing system. Figure S2: Relative humidity control system (Self-made system).
