# Supplementary material for: Development of High-Performance Ethanol Gas Sensors Based on La2O3 Nanoparticles-Embedded Porous SnO2 Nanofibers
Source: Sensors (Basel). 2024 Oct 24;24(21):6839. doi: 10.3390/s24216839 (PMC11548235; doi:10.3390/s24216839)
Supplement: Supplementary file 1 [file sensors-24-06839-s001.zip › sensors-3257767-supplementary.pdf]

sensors

MDPI

*Supplementary Materials*

# Development of High-Performance Ethanol Gas Sensors Based on $La_2O_3$ Nanoparticles-Embedded Porous $SnO_2$ Nanofibers

**Gen Li [1,†], Jian Hou [2,†], Muhammad Hilal [3], Hyojung Kim [3], Zhiyong Chen [2], Yunhao Cui [4], Jun-Hyun Kim [5,*] and Zhicheng Cai [3,*]**

[1] College of Computer Science, Chengdu University, Chengdu 610106, China; ligen@cdu.edu.cn
[2] School of Intelligent Manufacturing, Luoyang Institute of Science and Technology, Luoyang 471023, China; jhou@lit.edu.cn (J.H.); 1854@163.com (Z.C.)
[3] Department of Semiconductor System Engineering, Sejong University, 209 Neungdong-ro, Gwangjin-gu, Seoul 05006, Republic of Korea; hilal1991@sejong.ac.kr (M.H.); hyojungkim0912@sejong.ac.kr (H.K.)
[4] School of Mechatronics Engineering, Henan University of Science and Technology, Luoyang 471023, China; 00cyh00@163.com
[5] Department of Chemistry, Illinois State University, Normal, IL 61790-4160, USA
* Correspondence: jkim5@ilstu.edu (J.-H.K.); cai1121@sejong.ac.kr (Z.C.)
† These authors contributed equally to this work.

**Citation:** Li, G.; Hou, J.; Hilal, M.; Kim, H.; Chen, Z.; Cui, Y.; Kim, J.-H.; Cai, Z. Development of High-Performance Ethanol Gas Sensors Based on $La_2O_3$ Nanoparticles-Embedded Porous $SnO_2$ Nanofibers. *Sensors* **2024**, *24*, 6839. https://doi.org/10.3390/s24216839

Academic Editor: Sang Sub Kim

Received: 28 September 2024
Revised: 16 October 2024
Accepted: 23 October 2024
Published: 24 October 2024

**Copyright:** © 2024 by the authors. Licensee MDPI, Basel, Switzerland. This article is an open access article distributed under the terms and conditions of the Creative Commons Attribution (CC BY) license (https://creativecommons.org/licenses/by/4.0/).

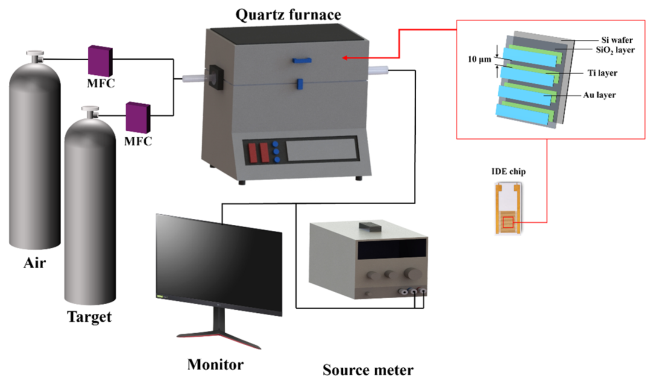


**Figure S1.** Schematic diagram of homemade gas sensing system.

To thoroughly investigate the sensor's response under varying relative humidity conditions, we designed a specialized system that integrates a Mass Flow Controller (MFC) with the sensing chamber. This setup enables precise control over both humidity levels and gas concentrations. The system includes four gas supply lines connected to the MFC for gas concentration regulation, a heated reservoir to adjust humidity, and a gas humidity sensor from S+S Regeltechnik. Among these, Line 5 introduces the target gas, while Lines 3 and 4 supply dry air. Line 1 is connected to the heated reservoir, with one end submerged in water and the other above it, allowing humid air to exit through Line 2. By merging Lines 2 and 3 under MFC regulation, a continuous flow of air with a set humidity is generated through the combination of dry and humid air. Line 4 is then integrated to adjust the airflow velocity, ensuring a stable output of air with the desired concentration and humidity. Finally, the mixed gas is directed through Line 5 to the sensing chamber (Line 6). Before entering the chamber, the gas's humidity and concentration are measured by the humidity sensor, providing real-time monitoring of the test gas properties.

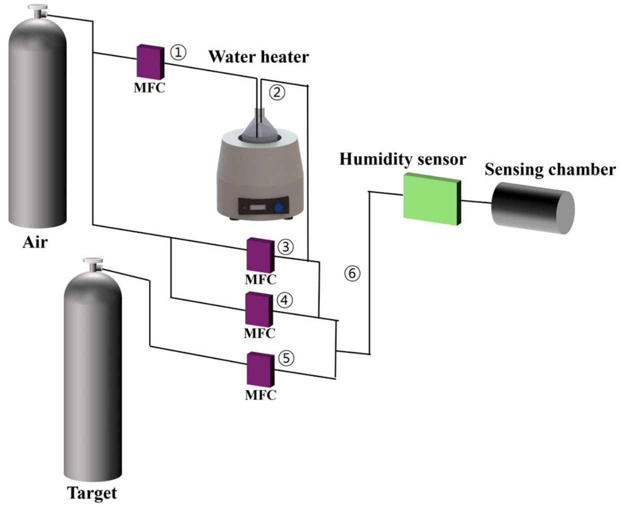


**Figure S2.** Relative humidity control system.

**Disclaimer/Publisher's Note:** The statements, opinions and data contained in all publications are solely those of the individual author(s) and contributor(s) and not of MDPI and/or the editor(s). MDPI and/or the editor(s) disclaim responsibility for any injury to people or property resulting from any ideas, methods, instructions or products referred to in the content.
